# Targeting Hypoxia in Cancer Cells by Restoring Homeodomain Interacting Protein-Kinase 2 and p53 Activity and Suppressing HIF-1α

**DOI:** 10.1371/journal.pone.0006819

**Published:** 2009-08-28

**Authors:** Lavinia Nardinocchi, Rosa Puca, Ada Sacchi, Gideon Rechavi, David Givol, Gabriella D'Orazi

**Affiliations:** 1 Department of Experimental Oncology, Molecular Oncogenesis Laboratory, National Cancer Institute “Regina Elena”, Rome, Italy; 2 Cancer Research Center, Chaim Sheba Medical Center, Tel-Hashomer and Sachler School of Medicine, Tel-Aviv University, Tel-Aviv, Israel; 3 Department of Molecular Cell Biology, Weizmann Institute of Science, Rehovot, Israel; 4 Department of Oncology and Neurosciences, University “G. d'Annunzio”, Chieti, Italy; Roswell Park Cancer Institute, United States of America

## Abstract

**Background:**

The tumor suppressor homeodomain-interacting protein kinase-2 (HIPK2) by phosphorylating serine 46 (Ser46) is a crucial regulator of p53 apoptotic function. HIPK2 is also a transcriptional co-repressor of hypoxia-inducible factor-1α (HIF-1α) restraining tumor angiogenesis and chemoresistance. HIPK2 can be deregulated in tumors by several mechanisms including hypoxia. Here, we sought to target hypoxia by restoring HIPK2 function and suppressing HIF-1α, in order to provide evidence for the involvement of both HIPK2 and p53 in counteracting hypoxia-induced chemoresistance.

**Methodology/Principal Findings:**

Upon exposure of colon and lung cancer cells to hypoxia, by either low oxygen or cobalt, HIPK2 function was impaired allowing for increased HIF-1α expression and inhibiting the p53-apoptotic response to drug. Cobalt suppressed HIPK2 recruitment onto HIF-1α promoter. Hypoxia induced expression of the p53 target MDM2 that downregulates HIPK2, thus MDM2 inhibition by siRNA restored the HIPK2/p53Ser46 response to drug. Zinc supplementation to hypoxia-treated cells increased HIPK2 protein stability and nuclear accumulation, leading to restoration of HIPK2 binding to HIF-1α promoter, repression of MDR1, Bcl2, and VEGF genes, and activation of the p53 apoptotic response to drug. Combination of zinc and ADR strongly suppressed tumor growth *in vivo* by inhibiting HIF-1 pathway and upregulating p53 apoptotic target genes.

**Conclusions/Significance:**

We show here for the first time that hypoxia-induced HIPK2 deregulation was counteracted by zinc that restored HIPK2 suppression of HIF-1 pathway and reactivated p53 apoptotic response to drug, underscoring the potential use of zinc supplementation in combination with chemotherapy to address hypoxia and improve tumor treatment.

## Introduction

Solid tumors can survive hypoxic condition (the high cell density of a tumor limits the availability of oxygen to cells) by using protective mechanisms including the activation of hypoxia-inducible factor-1α (HIF-1α) a transcription factor that induces, among others, antiapoptotic Bcl2, multidrug resistance (MDR), VEGF gene expression, and reprogramming of glucose metabolism that account for cell proliferation, angiogenesis, and chemoresistance [1]. Moreover, hypoxia attenuates the response of oncosuppressor p53 to cellular damage [2]. The p53 protein plays important roles in growth arrest, cellular repair, and cell death, which minimize the propagation of malignant cells [3]. The function of p53 as a tumor suppressor is linked to its activity as a transcription factor through posttranslational modifications that allow the protein to escape MDM2 control, accumulate, and become active [4]. The p53 gene is mutated in ∼50% of human cancers whereas, in cancers harbouring wild-type (wtp53), its activity may be compromised by other mechanisms including deregulation of regulatory proteins [5], [6].

Homeodomain-interacting protein kinase-2 (HIPK2) is an important regulator of p53 apoptotic function, thus we have previously shown that HIPK2 phosphorylates p53 at serine 46 (Ser46) after severe DNA damage, inducing p53 specific apoptotic transcriptional activity [7]–[9]. Phosphorylation at this site is a late event after severe DNA damage and specifically regulates p53-induced apoptosis through for instance upregulation of p53AIP1 gene instead of cell-cycle arrested related gene and MDM2 gene expression [10], [11]. A major auto-regulatory, negative feed-back loop of p53 involves p53-dependent MDM2 induction that in turn binds and inactivates p53 by driving it to proteasomal degradation [12]–[14]. In this regard, we have shown that HIPK2 neutralizes MDM2 inhibition rescuing p53 transcriptional activity and apoptotic function [15]. Therefore, agents such as HIPK2 that can increase active p53 in tumor cells by hinder the MDM2-p53 interaction might have therapeutic utility in sensitizing tumor cells to chemo- or radio-therapy.

HIPK2 is also a transcriptional co-repressor often in multiprotein complex with other co-repressors such as Groucho and hystone deacetylase 1 (HDAC1) [16]. We recently found that HIPK2 co-represses the hypoxia-inducible factor-1α (HIF-1α) transcription factor restraining HIF-1-induced tumor angiogenesis and chemoresistance [17]. Thus, inhibition of HIF-1α activity by HIPK2 reduces VEGF, MDR1, and Bcl2 expression and stimulates drug-induced apoptosis in p53-dependent and-independent ways [18]. Given its central role in the targeting of cells towards apoptosis upon genotoxic stress, the regulation of HIPK2 has been the subject to intense investigation in the last years. HIPK2 was found downmodulated in thyroid, breast [19] and colon cancers [20] in comparison to the respective normal tissues; mutated within the speckle retention signal in human acute myeloblastic leukemias and in myelodysplastic syndrome [21]; and delocalized in the cytoplasm by high-mobility group A1 (HMGA1) overexpression [22]. HIPK2 is an unstable protein that is degraded via the proteasome pathway in particular recent studies showed that HIPK2 can be downmodulated by p53-induced MDM2 [23] and by hypoxia-induced Siah2 proteins [24]. We have recently shown that HIPK2 knockdown induces p53 misfolding that can be reverted by zinc supplementation [25], [26]. Therefore, all the conditions that lead to HIPK2 deregulation would end in a multifactorial response leading to tumor chemoresistance by strongly affecting p53 transcriptional activity and apoptosis on one hand and HIF-1 activity on the other hand. Hence, an understanding of how downregulated HIPK2 could be reactivated might lead to new strategies to both restrain HIF-1 pathway and re-establish p53 activity to overcome drug resistance. This is also in line with recent works that have shown that treatment of cancer by anti-angiogenic agents may result in hypoxia that selects for radio and drug resistance, underscoring the need to address tumor hypoxia in cancer [27].

Zinc is an important cofactor for DNA-binding activity of p53, as some p53 mutations that perturb the zinc-binding site in p53 result in the loss of DNA binding [28]. Zinc is a trace element that is essential for the normal function of cells and is a cofactor for the structure and function of a wide range of cellular proteins including enzymes, transcription factors, and structural proteins [29]. Thus zinc is an essential prerequisite for the progress of many signalling processes in eukaryotes and deregulation of its metabolism can induce DNA damage and cancer risk [30]. Treatment with zinc was shown to have real clinic potential, reducing tumor growth and aggressiveness with limited biotoxicity, for instance in prostate cancer [31].

The purpose of our investigation was to evaluate whether hypoxia could deregulate the HIPK2-induced p53 dependent apoptotic transcriptional activity in response to drug and therefore contribute to chemoresistance and whether zinc could counteract this HIPK2/p53Ser46 inhibition. We found that exposure of tumor cells to hypoxia, by either low oxygen or cobalt, inhibited p53Ser46 phosphorylation and p53 apoptotic transcriptional activity in response to drug. This inhibition was the consequence of HIPK2 downregulation due, at least in part, to hypoxia-induced MDM2 upregulation. On the other hand, cobalt inhibited HIPK2 recruitment onto HIF-1α promoter. Notably, zinc supplementation to hypoxia-treated cells counteracted the inhibition of HIPK2 allowing its nuclear accumulation that correlated with HIF-1α downmodulation and repression of the HIF-1 pathway and with restoration of p53Ser46 apoptotic transcriptional activity in response to drug. Altogether, these results show that hypoxia-condition can affect the HIPK2/p53Ser46 pathway, notably we show here for the first time that zinc can neutralize the hypoxia-induced HIPK2 inhibition. Since tumors contain regions with hypoxic conditions where wtp53 is inactive, these results support the potential use of zinc supplementation to chemotherapy in treatment of tumors with non functioning wtp53 or HIPK2.

## Results

### Cell exposure to cobalt abrogates the p53 apoptotic-gene transcription and reduces HIPK2 recruitment onto HIF-1α promoter

**Figure 1:**
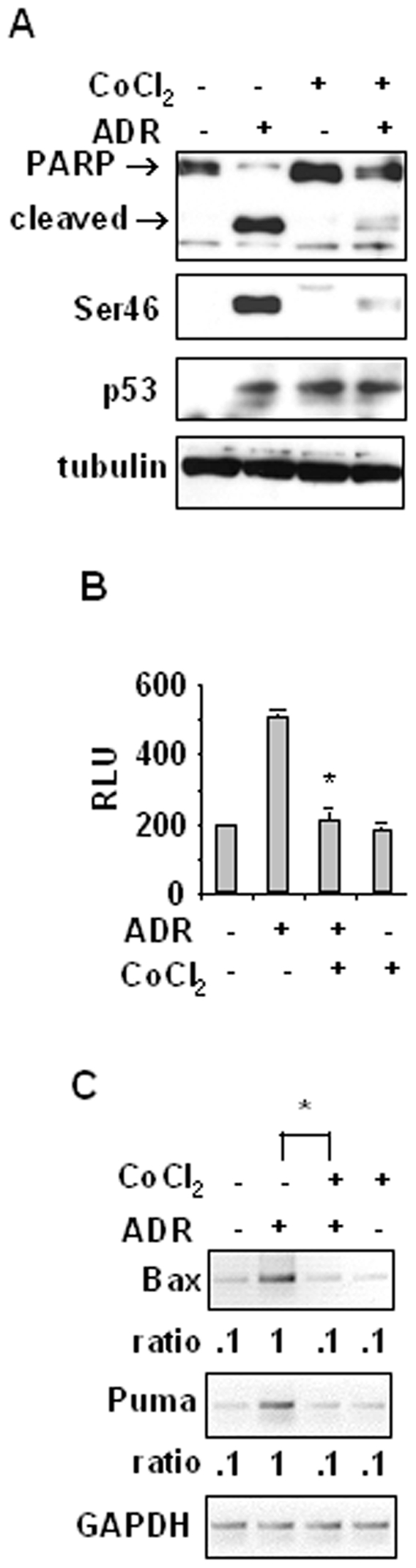
Cobalt inhibits the p53 apoptotic gene transcription in response to chemotherapy. (A) RKO cells were treated with CoCl_2_ and ADR for 16 h, alone or in combination. Equal amount of total cell extracts were analyzed by Western immunoblotting with specific antibodies detecting Ser46 phosphorylation and PARP cleavage (arrows: uncleaved and cleaved forms); total p53 is also shown. Anti-tubulin was used as protein loading control. (B) RKO cells, stably transfected with p53AIP1-luc reporter, were treated with CoCl_2_ and ADR for 16 h before luciferase activity was assayed. RLU: relative luciferase unit. *Columns*, mean of three independent experiments performed in duplicate; *bars*, S.D. * *P*<0.01. (C) Total mRNAs were reverse transcribed from RKO cells treated with CoCl_2_ and ADR alone or in combination, for 16 h for PCR analyses of p53 target genes *Bax* and *Puma*. GAPDH was used as internal control. Expression ratio to GAPDH was evaluated by densitometric analysis of gene expression. * *P*<0.01.

We asked whether cobalt chloride (CoCl_2_), that stabilizes HIF-1α and induces HIF-1 responsive genes with kinetics similar to that of hypoxia [32], could affect drug-induced p53 apoptotic gene transcription. As shown in Figure 1A, cobalt strongly abolished the ADR-induced PARP cleavage and Ser46 phosphorylation. Cobalt alone induced p53 levels, although it did neither induce Ser46 phosphorylation nor PARP cleavage (Figure 1A). The p53 apoptotic transcriptional activity was evaluated by luciferase assay. As shown in Figure 1B, the ADR-induced p53AIP1-luc activity was significantly impaired by cobalt, while cobalt alone did not affect it, and *in vivo* analysis of mRNA levels showed that drug-induced upregulation of p53 apoptotic target genes *Bax* and *Puma* was strongly impaired by cobalt (Figure 1C), as shown also by the expression ratio to GAPDH. Conversely, we had previously shown that cobalt can induce *MDR1*, and *Bcl2* gene expression [18].

**Figure 2:**
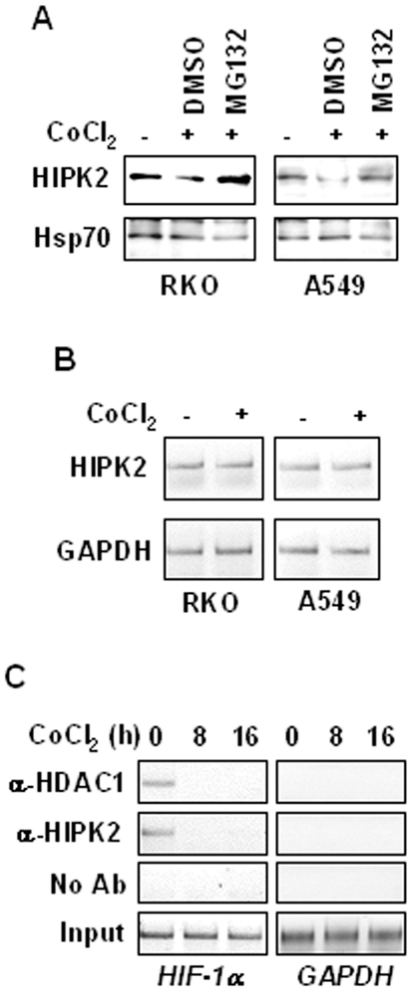
Cobalt downregulates HIPK2 protein levels and abrogates HIPK2 recruitment onto HIF-1α promoter. (A) RKO and A549 cells were treated with cobalt for 16 h in the presence or absence of proteasome inhibitor MG132 (40 µmol/L for 6 h) and the vehicle DMSO. Equal amount of total cell extracts were analyzed by Western immunoblotting with specific antibody detecting endogenous HIPK2 protein levels; anti-Hsp70 was used as protein loading control. (B) Total mRNAs were reverse transcribed from RKO and A549 cell treated with cobalt for 16 h for PCR analyses of HIPK2 gene expression. GAPDH was used as internal control. (C) Chromatin immunoprecipitation (ChIP) analysis performed with anti-HIPK2 and anti-HDAC1 antibodies on RKO cells treated with cobalt for 8 and 16 h. PCR analyses were performed on the immunoprecipitated DNA samples using specific primers for the human *HIF-1α* promoter. Amplification of *GAPDH* promoter (right panel) was used as control of HIPK2 binding specificity to the *HIF-1α* promoter. A sample representing linear amplification of the total input chromatin (Input) was included as control. Additional controls included immunoprecipitation performed with non-specific immunoglobulins (No Ab).

As hypoxia condition promotes at least in part HIPK2 protein degradation [24] we asked whether cobalt was able to similarly affect HIPK2 expression and activity. To this end, HIPK2 protein levels were examined in RKO and A549 cells treated with CoCl_2_ in the presence or absence of proteasome inhibitor MG132. As shown in Figure 2A, cobalt downregulated HIPK2 protein levels that could be rescued by MG132 treatment. Analysis of mRNA expression showed that HIPK2 gene transcription was not affected by cobalt (Figure 2B). We reasoned that by keeping HIPK2 protein levels low, cobalt could influence HIPK2 recruitment onto target promoters, thereby affecting HIPK2 co-repressor activity. In support of this hypothesis, we performed ChIP assay whereas chromatin immunocomplexes were immunoprecipitated with anti-HIPK2 and anti-Histone deacetylase 1 (HDAC1) antibodies and PCR analysis performed using specific primers flanking the HIF-1α promoter [17]. As shown in Figure 2C (left panel), the HIPK2 and HDAC1 recruitment onto *HIF-1α* promoter was strongly impaired by cobalt. As a control of HIPK2 binding specificity to the *HIF-1α* promoter, we used specific primers spanning the human *GAPDH* promoter region. As expected (Figure 2C, right panel), no *GAPDH* amplification was observed after chromatin immunoprecipitation with anti-HIPK2 and anti-HDAC1 antibodies while a clear PCR band was detected using the genomic DNA as template. Altogether, these results show that cobalt could affect both p53 and HIPK2 activity.

### Hypoxia-induced inhibition of p53 transcription activity can be reverted by MDM2 depletion

**Figure 3:**
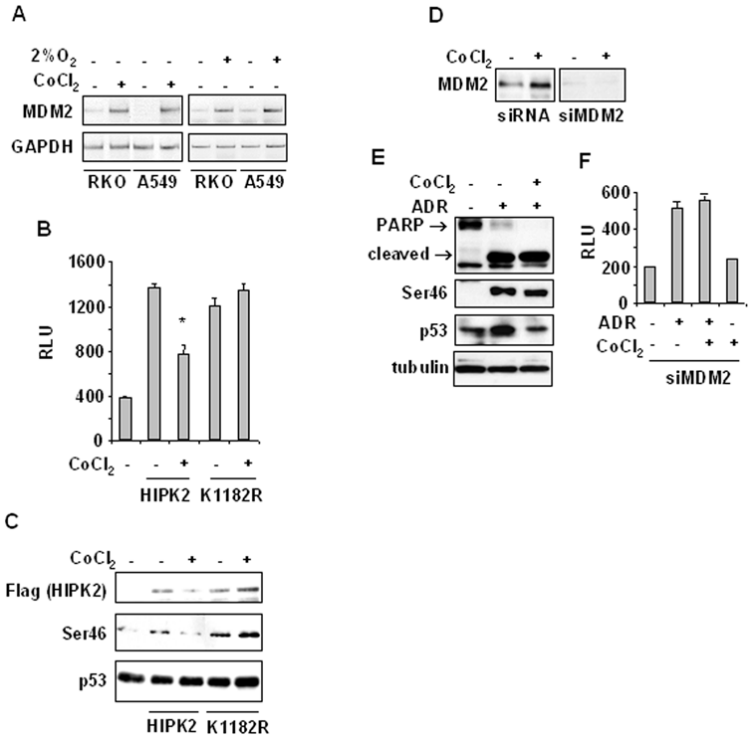
Up-regulation of MDM2 is the major inhibitor mechanism of p53 apoptotic transcription in hypoxia. (A) Total mRNAs were reverse transcribed from RKO and A549 cell treated with cobalt or 2% O_2_ for 16 h for PCR analyses of MDM2 gene expression. GAPDH was used as internal control. (B) 293 cells were co-transfected with p53AIP1-luc reporter and HIPK2-Flag or K1182R-Flag (MDM2 degradation-resistant mutant) expression vectors and 24 h later treated with CoCl_2_ for 16 h, before luciferase activity was assayed. RLU: relative luciferase unit. *Columns*, mean of three independent experiments performed in duplicate; *bars*, S.D. * *P*<0.01. (C) Cells were treated as in (B) and after treatment equal amounts of total cell extracts were subjected to Western immunoblotting using the indicated antibodies: anti-Flag (to detect ectopic HIPK2-Flag expression), anti-Ser46 and anti-p53 antibodies. (D) RKO cells were transfected with siMDM2 and 36 h later equal amount of total cell extracts were analyzed by Western immunoblotting with specific anti-MDM2 antibody. (E) RKO cells were transfected with siMDM2 and 24 h later were treated with CoCl_2_ and ADR for 16 h. Equal amount of total cell extracts were analyzed by Western immunoblotting with specific antibodies detecting p53Ser46 phosphorylation and PARP cleavage (arrows show cleaved and uncleaved forms); total p53 is also shown. Anti-tubulin was used as protein loading control. (F) RKO cells, stably transfected with p53AIP1-luc reporter, were transfected with siMDM2 and 24 h later treated with CoCl_2_ and ADR for 16 h before luciferase activity was assayed. RLU: relative luciferase unit. *Columns*, mean of three independent experiments performed in duplicate; *bars*, S.D.

HIPK2-mediated p53Ser46 phosphorylation is induced by severe DNA damage [7]–[9] that, in a feedback regulatory loop, strengthens the HIPK2 activity through a p53-mediated caspase-induced mechanism [33] and selectively induces apoptotic genes and inhibits the cell-cycle arrest-related and MDM2 genes [10], [11]. On the other hand, a non-severe stress can downregulate HIPK2 through p53-induced MDM2 upregulation [23]. Hence, we asked whether hypoxia could act as non-severe stress able to activate p53-target MDM2 and consequently inhibit HIPK2 and predispose to drug resistance. Semiquantitative RT-PCR analyses showed that cobalt as well as low oxygen induced MDM2 upregulation (Figure 3A). Next, we investigated whether MDM2 was responsible for inhibition of p53 apoptotic activity. Analysis of p53 transcriptional activity following cobalt treatment was performed in 293 cells co-transfected with the p53AIP1-luc reporter and either HIPK2-Flag or the HIPK2-K1182R-Flag point mutant that cannot be degraded by MDM2 [23]. As shown in Figure 3B, the HIPK2-induced AIP1-luc activity was significantly reduced by cobalt while the K1182R-induced AIP1-luc activity did not change. Western immunoblotting showed that HIPK2 expression was reduced by cobalt while the expression of the degradation-resistant K1182 mutant was not affected (Figure 3C). In agreement, also the HIPK2-induced Ser46 phosphorylation was reduced by cobalt, while it did not change following K1182 overexpression (Figure 3C). Both results suggested an involvement of MDM2 in HIPK2 regulation that in turn affected p53Ser46 activity. To test this hypothesis, RKO cells were depleted of the MDM2 function by siRNA (Figure 3D) and this MDM2 depletion was sufficient to rescue ADR-induced Ser46 phosphorylation and PARP cleavage inhibited by cobalt (Figure 3E and compare with 1A). Furthermore, MDM2 depletion counteracted the cobalt-induced inhibition of p53AIP1-luc activity in response to ADR (Figure 3F and compare with 1B). Altogether, these data suggest that the hypoxia-induced drug resistance was dependent, at least in part, on upregulation of MDM2 expression that in turn inhibited HIPK2/p53Ser46 apoptotic activity in response to drug.

### Zinc restores ADR-induced p53 apoptotic-gene transcription in cobalt-treated cells

**Figure 4:**
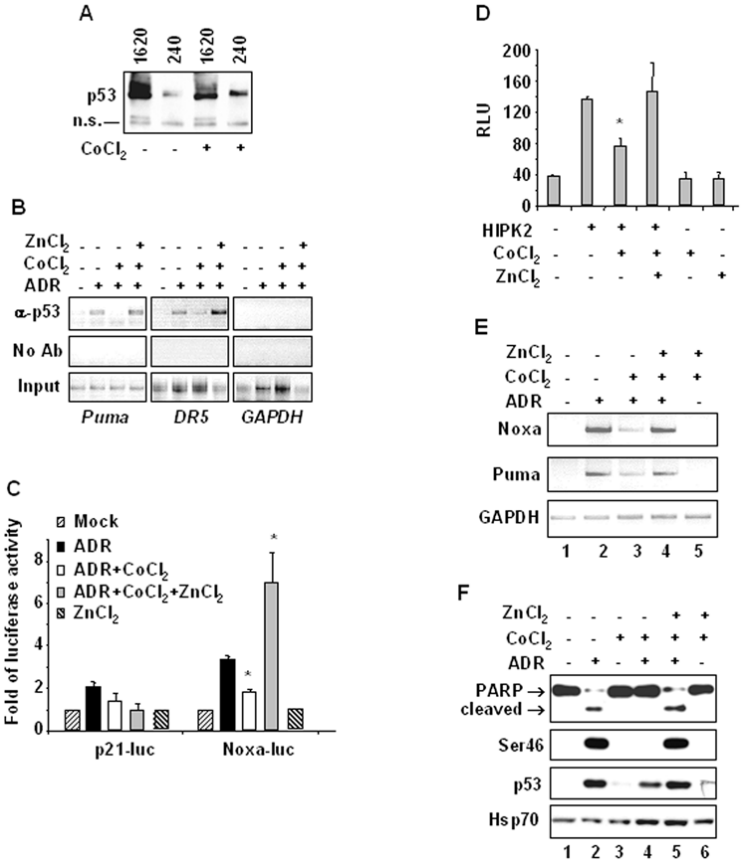
Effect of zinc on reversing hypoxia-induced inhibition of p53Ser46 in response to chemotherapy. (A) RKO cells were treated with cobalt for 16 h and equal amount of total cell extracts were immunoprecipitated with conformation-specific Pab1620 (for wild-type, folded p53 form) and Pab240 (for mutant, unfolded p53 form) antibodies and analyzed by Western immunoblotting with anti-p53 polyclonal antibody (FL393). The representative bands from at least two independent experiments are presented, showing increase of PAb240 reactivity and reduction of Pab1620 reactivity after cobalt treatment. A non specific (n.s.) signal is shown as protein loading control. (B) Chromatin immunoprecipitation (ChIP) analysis performed with anti-p53 antibody on RKO cells exposed to ZnCl_2_ and CoCl_2_ for 24 h and Adriamycin for 16 h. PCR analyses were performed on the immunoprecipitated DNA samples using specific primers for p53 target *Puma* and *DR5* promoters. Amplification of *GAPDH* promoter (right panel) was used as control of p53 binding specificity to *Puma* and *DR5* promoters. A sample representing linear amplification of the total input chromatin (Input) was included as control. Additional controls included immunoprecipitation performed with non-specific immunoglobulins (No Ab). (C) RKO cells were transfected with p21-luc and Noxa-luc reporters and 24 h later treated with ZnCl_2_ and CoCl_2_ for 24 h and Adriamycin for 16 h respectively, before luciferase activity was assayed. Results, normalized to β-galactosidase activity are shown as fold of induction over untreated cells; *bars*, S.D. (D) 293 cells were co-transfected with p53AIP1-luc reporter and HIPK2-Flag expression vector and 24 h later treated with ZnCl_2_ and CoCl_2_ for 16 h, before luciferase activity was assayed. RLU: relative luciferase unit. *Columns*, mean of three independent experiments performed in duplicate; *bars*, S.D. * *P*<0.01. (E) Total mRNAs were reverse transcribed from RKO cells treated as in (C) for PCR analyses of p53 target genes *Noxa* and *Puma*. GAPDH was used as internal control. (F) RKO cells were treated with ZnCl_2_ and CoCl_2_ for 24 h and ADR for 16 h and equal amount of total cell extracts analyzed by Western immunoblotting with anti-PARP (arrows show the uncleaved and cleaved PARP), anti-Ser46 and anti-p53 antibodies. Anti-Hsp70 was used as protein loading control.

We have recently shown that HIPK2 depletion is responsible for p53 protein misfolding that can be reverted by zinc supplementation [25], [26]. Hence, we hypothesized here that hypoxia-induced HIPK2 deregulation might mirror the HIPK2 knockdown condition and thereby affect p53 DNA-binding and transcriptional activities. We found that cobalt increased the p53 reactivity to the PAb240 antibody (mutant, unfolded p53 form) and reduced the p53 reactivity to the Pab1620 antibody (wild-type, folded form) (Figure 4A) indicating p53 protein misfolding, and therefore tested whether zinc supplementation was able to restore wtp53 activity in response to drug. To this aim we first evaluated the *in vivo* wtp53 DNA-binding activity by using ChIP analysis. RKO cells were treated with cobalt and ADR in the presence or absence of zinc and endogenous p53 immunorecipitated with polyclonal anti-p53 antibody (FL393). The amount of co-precipitated p53-binding elements in target promoters was determined by PCR. The results showed that cobalt markedly reduced p53 binding to promoters of apoptotic genes like *Puma* and *DR5* in response to ADR and that this inhibition was strongly reverted by zinc supplementation (Figure 4B). The p53 specific binding to *Puma* and *DR5* promoters was confirmed by *GAPDH* promoter amplification after chromatin immunoprecipitation with anti-p53 antibody (Figure 4B). Thus, p53 apoptotic transcriptional activity specifically induced by ADR (Noxa-luc versus p21-luc), was inhibited by cobalt and restored by zinc supplementation (Figure 4C). Notably, zinc alone did not induce p53 transcriptional activity. Furthermore, we evaluated whether HIPK2-induced p53 transcriptional activity inhibited by cobalt (Figure 3B) could be restored by zinc. To this aim, 293 cells were co-transfected with p53AIP1-luc reporter and HIPK2 expression vector. As shown in Figure 4D, zinc supplementation fully restored HIPK2-induced p53AIP1-luc activity reduced by cobalt, while treatments with cobalt or zinc alone did not induce p53AIP1 luciferase activity. In agreement, the ADR-induced p53 apoptotic gene transcription was inhibited by cobalt (Figure 4E, compare lane 2 with lane 3) and restored by zinc supplementation (Figure 4E, compare lane 3 with 4) to the same levels of ADR treatment (Figure 4E compare lane 4 with lane 2). Notably, zinc supplementation to cobalt did not induce apoptotic gene transcription (Figure 4E, lane 5). Finally, apoptotic cell death was evaluated by Western immunoblotting showing that inhibition of PARP cleavage and Ser46 phosphorylation by cobalt exposure of ADR-treated cells (Figure 4F, compare lane 2 with lane 4) was strongly restored by zinc (Figure 4F, compare lane 4 with lane 5). These data suggest that zinc was able to reactivate the hypoxia-inhibited endogenous wtp53 DNA-binding and apoptotic transcriptional activities in response to drug, by counteracting, at least in part, the HIPK2 deregulation.

### Zinc reverts dysfunctional HIPK2 in hypoxia-treated cells

**Figure 5:**
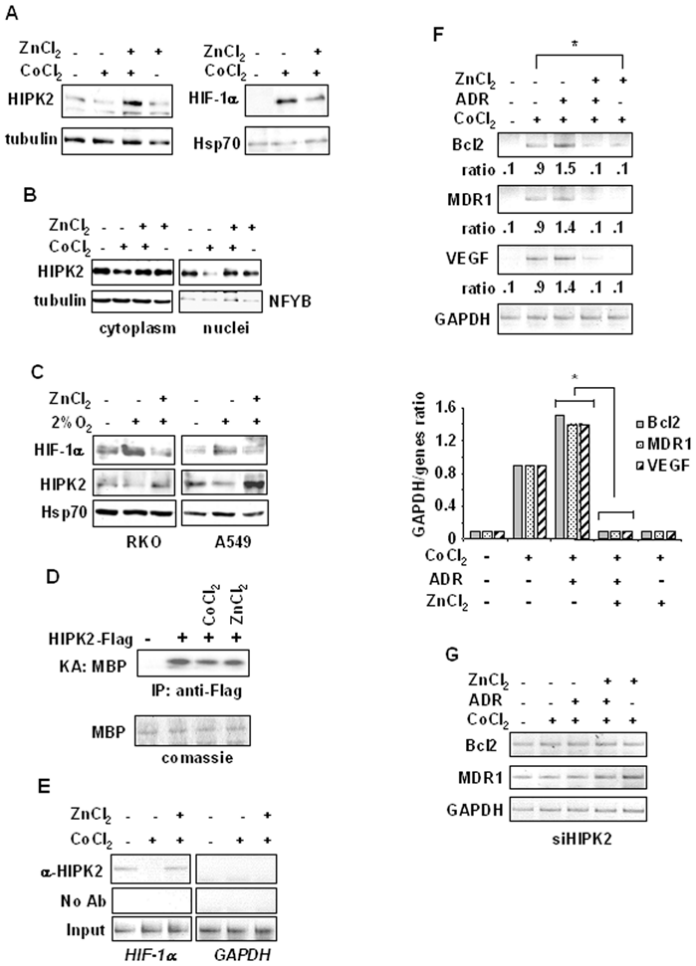
Effect of zinc on reversing hypoxia-induced HIPK2 dysfunction and protecting against hypoxia pathway. (A, left panel) Subconfluent RKO cells were exposed to CoCl_2_ and ZnCl_2_ alone or in combination for 16 h, and equal amount of total cell extracts analyzed by Western immunoblotting with specific antibody showing endogenous HIPK2 level. Anti-tubulin was used as protein loading control (right panel). Equal amount of nuclear extracts of RKO cells treated as above, were analyzed by Western immunoblotting with specific antibodies detecting HIF-1α levels. Anti-Hsp70 was used as protein loading control. (B) RKO cells treated as in (A) were lysed for nuclear and cytoplasmic fractionation and analyzed by Western immunoblotting using anti-HIPK2 antibody. Anti-tubulin and anti-NFYB antibodies were used for protein loading control of cytoplasmic and nuclear fractions, respectively. (C) RKO and A549 cells were treated with 2% O_2_ in the presence or absence of zinc for 16 h, and equal amount of nuclear cell extracts analyzed by Western immunoblotting with specific antibodies detecting HIF-1α and HIPK2 nuclear levels. Anti-Hsp70 was used as protein loading control. (D) Kinase assay of ectopic HIPK2 in 293 cells transfected with Flag empty or HIPK2-Flag expression vector left untreated or treated with cobalt or zinc for 16 h. Equal amount of total cell extracts were immunoprecipitated using anti-Flag antibody and assayed for kinase activity using MBP as substrate. Equal expression of MBP protein was confirmed by comassie-staining of kinase assay. (E) Chromatin immunoprecipitation (ChIP) analysis performed with anti-HIPK2 antibody on RKO cells treated as in (A). PCR analyses were performed on the immunoprecipitated DNA samples using specific primers for the human *HIF-1α* promoter. Amplification of *GAPDH* promoter (right panel) was used as control of HIPK2 binding specificity to the *HIF-1α* promoter A sample representing linear amplification of the total input chromatin (Input) was included as control. Additional controls included immunoprecipitation performed with non-specific immunoglobulins (No Ab). (F, upper panel), Total mRNAs were reverse transcribed from RKO cells treated with ZnCl_2_ and CoCl_2_ for 24 h and ADR for 16 h respectively, for PCR analyses of HIF-1 target genes *Bcl2*, *MDR1*, and *VEGF*. Ratio: expression ratio to GAPDH. (F, lower level) densitometric analysis of gene expression plotted as the expression ratio to GAPDH, used as internal control. Student's *t* test was used for statistical analysis of comparison between the values of cobalt and cobalt plus zinc, and of CoCl_2_/ADR and CoCl_2_/ADR/ZnCl_2_ as shown. * *P*<0.01. (G) RKO cells depleted of HIPK2 function by siHIPK2 were treated with ZnCl_2_ and CoCl_2_ for 24 h and ADR for 16 h, respectively and total mRNAs reverse transcribed for PCR analyses as in (F).

Next we sought to evaluate whether zinc could counteract hypoxia-induced HIPK2 downregulation and affect HIPK2 binding to chromatin. As shown in Figure 5A (left panel) the cobalt-induced HIPK2 protein downregulation was rescued by zinc supplementation while zinc alone did not change the HIPK2 levels. Conversely, cobalt treatment upregulated HIF-1α expression and this effect was robustly reverted by zinc supplementation (Figure 5A, right panel). Subcellular fractionation showed that cobalt treatment reduced both cytoplasmic and nuclear HIPK2 nuclear levels, although HIPK2 nuclear levels were more strongly affected by cobalt and that this effect was reverted by zinc supplementation (Figure 5B). Zinc alone did not affect HIPK2 levels (Figure 5B). Similar results were obtained with low oxygen treatment (Figure 5C). We next analysed HIPK2 catalytic activity after cobalt treatment. To this aim, 293 cells were transfected with Flag empty vector or HIPK2-Flag expression vector in the presence or absence of cobalt or zinc, followed by immunoprecipitation of ectopic HIPK2 with anti-Flag antibody and *in vitro* kinase assay with the known HIPK2 phosphorylation substrate myelin basic protein (MBP) [7]. As shown in Figure 5D, ectopic HIPK2 was still able to phosphorylate MBP after treatments, suggesting that hypoxia likely does not affect HIPK2 catalytic activity, at least in our experimental condition.

The opposite effects of the hypoxia on HIPK2 and HIF-1α levels are interconnected by the repressor effect of HIPK2 on *HIF-1α* promoter. Thus, the cobalt-induced abolishment of HIPK2 recruitment onto *HIF-1α* promoter was strongly reverted by zinc (Figure 5E). The HIPK2 specific binding to *HIF-1α* promoter was confirmed by *GAPDH* promoter amplification after chromatin immunoprecipitation with anti-p53 antibody. Consequently, RT-PCR analysis showed that the cobalt-induced *Bcl-2*, *MDR1* and *VEGF* gene expression was significantly suppressed by zinc (Figure 5FD, compare CoCl_2_ lanes with CoCl_2_/ZnCl_2_ lanes), as also shown by the expression ratio to GAPDH (Figure 5F, lower panel). Moreover, ADR treatment in combination with cobalt was not able to inhibit the cobalt-induced HIF-1 pathway (Figure 5E, compare CoCl_2_ lanes with CoCl_2_/ADR lanes) unless in combination with zinc (Figure 5F, compare CoCl_2_/ADR lanes with CoCl_2_/ADR/ZnCl_2_ lanes). The role of HIPK2 in cobalt-induced HIF-1 targets was further evaluated following HIPK2 depletion. As shown in Figure 5G, the basal level of *MDR1* and *Bcl2* gene expression was already high in siHIPK2 cells, in agreement with our previous results on HIPK2 repressor activity of HIF-1α [17], [18], and could not be repressed by zinc treatment (Figure 5G and compare with 5F). The effect of zinc counteracting hypoxia outcome was specific as another antioxidant, i.e., vitamin C, did neither downmodulate HIF-1 target genes nor affected drug response (data not shown). Altogether, these results show that zinc counteracted hypoxia-induced HIPK2 downmodulation, recovered the HIPK2 recruitment onto chromatin and led to repression of the HIF-1 pathway.

### Zinc improves chemotherapeutic response in vivo

**Figure 6:**
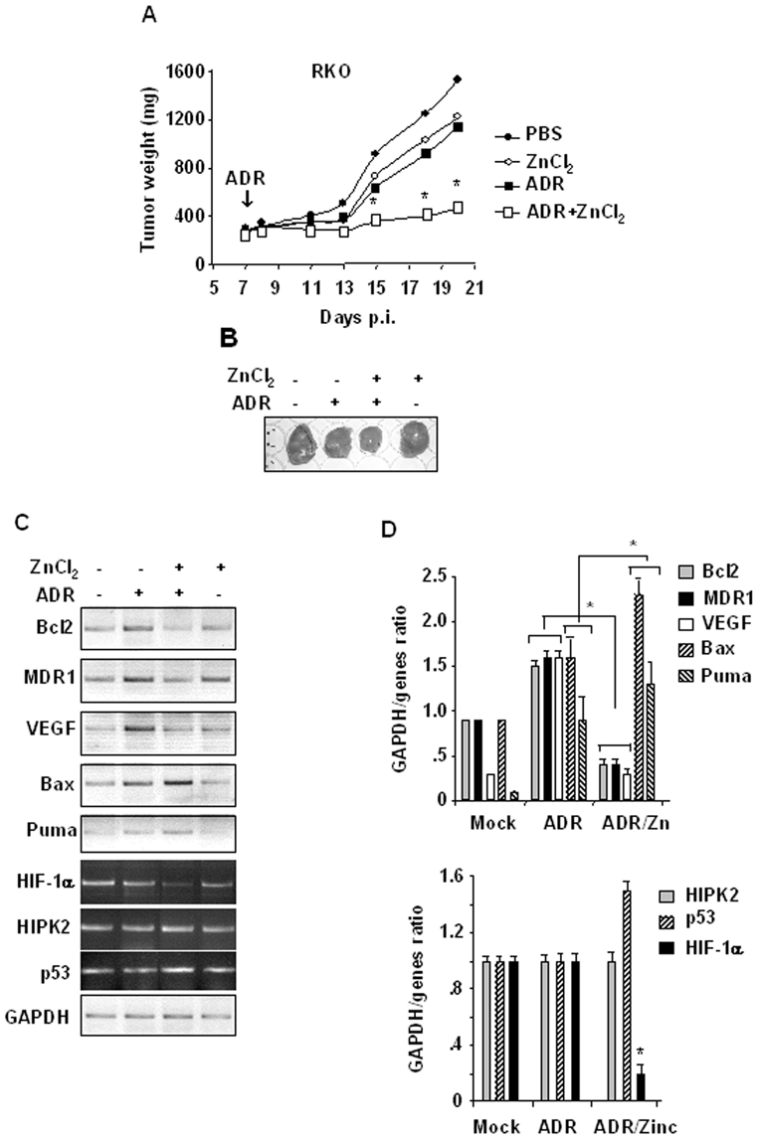
Therapeutic efficacy of zinc combined with chemotherapy. (A) RKO cells were implanted into nude mice by i.m. injection and allowed to develop into palpable ∼300 mm^3^ tumor nodules at the injection sites and the randomized to four treatment groups: (*i*) vehicle (PBS) alone, (*ii*) ADR alone (10 mg/kg body weight) delivered once intraperitoneally (i.p.) at day seven (arrow), (*iii*), ZnCl_2_ alone (10 mg zinc/kg body weight) administrated once daily by oral administration, and (*iv*) ZnCl_2_ combined with chemotherapy: tumors were pre-treated at day 7 with ZnCl_2_ for 8 h before delivering ADR and thereafter, ZnCl_2_ was administered once daily for the next 2 weeks. Each experiment was conducted two independent times, each time with eight mice per group. Tumor volumes were measured every other day following the establishment of xenografts in mude mice. *Y axis*, tumor volume; *X axis*, calibration time (days) after cell injection (p.i.: post-injection). Student's *t* test was used for statistical analysis of comparison between the values of ADR and ADR in combination with zinc treatments, as shown. * *P*<0.01. (B) Picture of explanted tumors showing tumor volumes at day 18. (C) RNA samples from explanted tumors, at day 18 after cell injection, treated with ADR and zinc alone or in combination were used for reverse-transcription (RT)-PCR. The mRNA levels were normalized to GAPDH expression. (D) Densitometric analysis of gene expression plotted as expression ratio to GAPDH. Average of two different tumors for each treatment group. Student's *t* test was used for statistical analysis of comparison between the values of ADR and ADR in combination with zinc treatments, as shown. * *P*<0.01.

To evaluate the therapeutic efficacy of the combination of zinc and chemotherapy *in vivo* we generated tumor xenografts in athymic nude mice. The mice were then pre-treated with ZnCl_2_ for 8 h before ADR injection and then treated every day with zinc. Mice treated with ADR alone over the course of 2 weeks displayed reduction of tumor volume in a comparable manner with those treated with zinc alone (Figure 6A). Interestingly, addition of zinc significantly enhanced the effect of ADR leading to marked inhibition of tumor growth (Adriamycin + zinc *versus* Adriamycin: *P*<0.01) (Figure 6A and 6B). Tumors were harvested by day 18 and gene expression was determined by RT-PCR and densitometric analyses. The results showed that the p53 target genes, including *Puma* and *Bax*, were induced by ADR and further increased by zinc supplementation; on the other hand, although *MDR1*, *Bcl2*, and *VEGF* genes were induced by ADR they were strongly downmodulated by combination of zinc and ADR (Figure 6C and 6D). Of note, while *HIPK2* and *p53* levels did not vary in the different tumor treatments, *HIF-1α* levels were significantly downmodulated by zinc combined with ADR (Figure 6C and 6D), strongly supporting our hypothesis of HIPK2 effect on HIF-1α transcription. Taken together, these data show that *in vivo* zinc supplementation improved tumor drug response, at least in part, by inhibiting the HIF-1 pathway and further activating the p53-dependent apoptosis similarly to the results obtained *in vitro*.

## Discussion

In this study we showed that hypoxia, by either low oxygen or cobalt, could downregulate HIPK2 expression and induce chemoresistance. The mechanistic explanation of hypoxia-induced chemoresistance involved upregulation of HIF-1 pathway and inhibition of the p53 pathway that were partly interconnected by the hypoxia-induced HIPK2 deregulation. Thus, in our cell lines, hypoxia, by inducing p53 target MDM2 could downregulate HIPK2 leading on the one hand to de-repression of the HIF-1α activity and on the other hand to inhibition of p53 apoptotic activity in response to drug. Of note, HIPK2 catalytic activity did not change after cobalt treatment, therefore suggesting that HIPK2 nuclear disappearance is the major reason of HIPK2 deregulation, at least in our experimental condition. Indeed, lack of HIPK2 nuclear accumulation during hypoxia, as seen in Figure 5B, and likely of interaction with other proteins, can mechanistically explain inhibition of HIPK2 co-repressor function on DNA, nonetheless HIPK2 keeps its kinase activity. Interestingly, we provided evidence here for the first time that zinc counteracted the hypoxia-induced HIPK2 downregulation and re-established HIPK2 recruitment to HIF-1α promoter with repression of HIF-1 target genes; notably, zinc restored p53/Ser46 apoptotic response to drug inhibited by hypoxia. Therefore, the therapeutic efficacy of zinc in combination with chemotherapy was evaluated *in vivo* in a tumor xenografts model, where zinc in combination with chemotherapy suppressed the HIF-1α pathway, improved p53 apoptotic transcription in response to drug, and strongly reduced tumor growth.

The p53 pathway is crucial for effective tumor suppression in humans and the state of p53 activity sets up life or death decision for the cell, as well as response to chemotherapy. There is a growing interest in the therapeutic possibilities of reactivating dysfunctional p53 in cancers in order to halt tumor growth. Obviously this requires understanding how p53 function was lost in the first place as p53 can be either mutated or deregulated by alteration of regulatory proteins. HIPK2 is a positive regulator of p53 that phosphorylates p53 at Ser46 endowing it with apoptotic activity [7], [34]. Thus, HIPK2 depletion inhibits p53 apoptotic activation and leads to chemoresistance [8], [25]. Of note, ectopic HIPK2 can restore p53/Ser46 apoptotic activity and overcome chemoresistance, as shown in an ovarian cancer model resistant to cisplatin [35]. These observations were confirmed by an independent approach of RNA-mediated gene silencing that identifies HIPK2 as one potential target for cellular resistance to chemotherapy [36]. Furthermore, we have recently shown that HIPK2 knockdown is responsible for misfolding of wtp53 that inhibits p53 DNA binding and transcriptional activity and that this outcome can be reverted by zinc supplementation affecting p53 conformation [25], [26]. Therefore, HIPK2 function/dysfunction can severely influence p53 oncosuppressor activity.

It has been shown that HIPK2 is activated by severe DNA damage [7], [8], [34], [37], [38] that induces p53Ser46 phosphorylation that, in a feedback regulatory loop, strengthens the HIPK2 activity through a p53-mediated caspase-induced mechanism [33] and selectively induces apoptotic genes while inhibits the cell-cycle arrest-related p21 and MDM2 genes [10], [11]. On the other hand, a non-severe DNA damage can downregulate HIPK2 through p53-induced MDM2 that degrades HIPK2 via proteasome [23]. In this study, we found that hypoxia, by functioning as a non-severe DNA damage, could induce MDM2 upregulation that was responsible, at least in part, for HIPK2 downregulation, as evidenced by recovering of HIPK2 accumulation after treatment with proteasome inhibitor MG132 (Figure 2A). MDM2 acts as a survival factor in many cell types and is overexpressed in a subset of human tumors where it is thought to contribute to the development of resistance to radiation and chemo-therapy [39]. The mechanistic explanation of MDM2-induced tumor chemoresistance was confirmed by MDM2 depletion with siRNA that was sufficient to rescue ADR-induced p53Ser46 phosphorylation and PARP cleavage inhibited by cobalt (Figure 3E). This was consistent with the finding that in contrast to HIPK2, the HIPK2 mutant K1182R, which is MDM2-resistant (23), was not affected by hypoxia-induced downregulation (Figure 3C) nor was its activity inhibited as shown in the K1182R-induced p53AIP1-luc activity (Figure 3B).

HIPK2 has an important role as a negative regulator of gene expression [40] and its elimination from promoter-associated repressor complexes allows for induction, for instance, of a substantial fraction of hypoxia-induced genes [24]. HIF-1 is an heterodimeric transcription factor that transactivates more than 60 target genes involved in multiple aspects of tumorigenesis including tumor growth, angiogenesis. metastasis, glucose metabolism and chemotherapy response. HIF-1 consists of two subunits, HIF-1α and HIF-1β [1]: HIF-1β is constitutively expressed in cells, while HIF-1α stability is stimulated by hypoxia, growth factors, and several oncogenes. A high level of HIF-1α is associated with pro-survival of the cancer cell, increased tumor angiogenesis, invasiveness, and resistance to conventional treatments [1], [32]. HIF-1α is mostly regulated at posttranscriptional levels by low oxygen condition, however we found that HIPK2 can transcriptionally regulate HIF-1α gene independently of the oxygen condition [17]. Thus, HIPK2 inhibition by RNA interference induces HIF-1α up-regulation and tumor angiogenesis [17]. On the other hand, inhibition of HIF-1α activity by HIPK2 reduces VEGF, MDR1, and Bcl2 expression and stimulates drug-induced apoptosis in p53-dependent and-independent ways [18], providing a rationale for the potential use of HIPK2 transduction or restoration of HIPK2 function to inhibit HIF-1 pathway and sensitize chemoresistant tumor cells to drugs. To the best of our knowledge, our finding that zinc might counteract the hypoxia-induced HIPK2 downregulation is the first attempt to try to restore HIPK2 activity, although the exact mechanism that leads to recovery of HIPK2 function by zinc remains to be elucidated. Zinc is a cofactor for the structure and function of a wide range of cellular enzymes, transcription factors including p53, zinc fingers and structural proteins [29]. We hypothesize that zinc might stabilize HIPK2 counteracting the MDM2- or hypoxia-dependent HIPK2 proteasomal degradation and increase HIPK2 sumoylation and transcriptional repressor function. In this regard, SUMO has been shown to act as antagonist of ubiquitination and have a role in the negative regulation of transcription, hence, sumoylation might block alternative lysine-targeted modifications such as acetylation or ubiquitination [41]. HIPK2 harbours a SUMO-interaction motif in its C-terminus that might affect its intramolecular conformation [42], and SUMO modification of HIPK2 has been shown to enhances it corepressor activity [43]. In agreement with these studies we found that zinc could increase HIPK2 nuclear accumulation and recruitment onto HIF-1α promoter, inhibited by hypoxia (Figure 5).

Zinc restores p53 function in HIPK2 depleted cells, as previously reported by our studies [25], [26], but can also affect HIPK2 function, as suggested by this study, and therefore be used as HIF-1α-targeting approach rendering the chemotherapeutic treatment of tumors more effective. Very few studies report the use of zinc in combination with chemotherapy, in fact as far as we know, zinc is not administered as part of any modern chemotherapy program in the treatment of cancer. It was shown that treatment with zinc alone can have real clinic potential, reducing tumor growth and aggressiveness with limited biotoxicity, for instance in prostate cancer [31]. Interestingly, one medical hypothesis reported that zinc adjuvant can accelerate recovery from acute lymphocytic leukaemia (ALL), and in conjunction with chemotherapy, cure ALL [44]. Therefore, whether zinc supplementation can aid in stimulating T-cell system to fight off tumors, as suggested by Eby's hypothesis [44] or whether it can affect gene expression pathways, such as HIF-1 and p53 pathways, to improve tumor response to drugs is a fascinating research field in cancer therapy that deserves further studies. Moreover, our preclinical finding of zinc and drug combination therapy for maximal therapeutic response of cancer cells to drugs might be of general importance in light of recent work showing that treatment of cancer by anti-angiogenic agents like anti VEGFR or small molecule inhibitors of tyrosine kinase may result in hypoxia that selects for more malignant metastatic and invasive cells, that eventually lead the tumors to relapse as a more invasive and metastatic disease [45], [46]. Similar results may apply also for the treatment with antibodies when the signaling pathway triggered by the antibody affects the HIF 1 pathway [47]. Hence, the more proficient an antiangiogenic agent is, the more efficiently it will cause hypoxia and unlike normal cells, tumor cells are much better equipped to cope with hypoxia. These surprising new results call upon new methods for targeting the hypoxia pathway in combination with the anti-angiogenic targeting drug. Thus, targeting HIF-1 is expected to synergize with the anti-angiogenesis treatment and other treatments that promote hypoxia and restore sensitivity to drugs. Our result provides a new answer to this surprising undesirable effect of the hypoxia on the final outcome of cancer in treated patients.

## Materials and Methods

### Cell lines and treatments

RKO human colon carcinoma, RKO-HIPK2-depleted [8], RKO-AIP1-luc (stable transfected with p53AIP1-luc vector) [48] and A549 human lung cancer cells were maintained in RPMI-1640 (Life Technology-Invitrogen) and human embryo kidney 293 cells were maintained in DMEM (Life Technology-Invitrogen), supplemented with 10% heat-inactivated fetal bovine serum plus glutamine and antibiotics in humidified atmosphere with 5% CO_2_ at 37°C.

For treatments, zinc chloride (ZnCl_2_) and Adriamycin (ADR) were added to the culture medium to a final concentration of respectively 100 µmol/L and 1.5 µg/mL for the indicated period; hypoxia condition was obtained by adding cobalt chloride (CoCl_2_) into the culture medium to a final concentration of 200 µmol/L for the indicated time or by exposing cell culture to 2% oxygen (O_2_) for 16 h. Proteasome inhibitor MG132 (Biomol, Research Laboratories) was diluted in DMSO at 40 mmol/L and added to the culture medium to a final concentration of 40 µmol/L for 6 h.

### RNA extraction and reverse transcription (RT)-PCR analysis

Cells and tumors were harvested in TRIzol Reagent (Invitrogen) and total RNA was isolated following the manufacturer's instructions. The first strand cDNA and the semi-quantitative RT-PCRs were carried out essentially as described [25] by using genes specific oligonucleotides under conditions of linear amplification. PCR was performed in duplicate in two different sets of cDNA. PCR products were run on a 2% agarose gel and visualized by ethidium bromide staining using UV light. The housekeeping glyceraldehydes-3-phosphate dehydrogenase (GAPDH) mRNA, used as internal standard, was amplified from the same cDNA reaction mixture. Densitometric analysis was applied to quantify mRNA levels. Data presented are representative of al least three independent experiments. Primer sequences are available upon request.

### Western immunoblotting and p53 immunoprecipitation

Total cell extracts were prepared by incubating at 4°C for 30 min in lysis buffer (50 mmol/L Tris-HCl (pH 7.5), 50 mmol/L NaCl, 5 mmol/L EDTA, 150 mmol/L KCl, 1 mmol/L DTT, 1% NP40) and a mix of protease inhibitors (Sigma) and resolved by SDS-polyacrylamide gel electrophoresis. Nuclear extracts were prepared essentially as described [18]. Proteins were transferred to a polyvinylidene difluoride (PVDF) membrane (Millipore) and incubated with the primary antibodies followed by an anti-IgG-horseradish peroxidase antibody (Bio-Rad). Immunoblotting was performed with: mouse monoclonal anti-p53 (DO1), mouse monoclonal anti-MDM2 (Ab1) (both from Santa Cruz Biotechnology), rabbit polyclonal anti-phospho-Ser46, (Cell Signaling Technology), rabbit polyclonal anti-HIPK2 (kindly provided by M.L. Schmitz, Justus-Liebig-University, Giessen, Germany), mouse monoclonal anti-poly(ADP-ribose) polymerase (PARP, BD Pharmingen), mouse monoclonal anti-HIF-1α (Novus Biologicals), mouse monoclonal anti-tubulin (Immunological Sciences), rabbit polyclonal anti NFYB (kindly provided by R. Mantovani, University of Milan, Italy) and mouse monoclonal anti-Hsp70 (Stressgene). Immunoreactivity was detected by enhanced chemiluminescence kit (ECL; Amersham).

For p53 conformation studies, p53 immunoprecipitation was performed essentially as described [25]. Briefly, 150–250 µg of total cell extracts were immunoprecipitated with conformation-specific monoclonal PAb240 (mutant specific) and PAb1620 antibodies (Calbiochem). Immunocomplexes were separated by 10% SDS-PAGE and immunoblotting was performed with rabbit anti-p53 antibody (FL393) (Santa Cruz Biotechnology). Immunoreactivity was detected with the ECL-chemoluminescence reaction kit (Amersham).

### Chromatin Immunoprecipitation (ChIP) assay

Chromatin Immunoprecipitation (ChIP) analysis was carried out essentially as described [48]. Protein complexes were cross-linked to DNA in living cells by adding formaldehyde directly to the cell culture medium at 1% final concentration. Chromatin extracts containing DNA fragments with an average size of 500 bp were incubated overnight at 4°C with milk shaking using rabbit polyclonal anti-HIPK2 (Santa Cruz Biotechnology), rabbit polyclonal anti-p53 (FL393; Santa Cruz Biotechnology), or rabbit polyclonal anti-Histone deacetylase 1 (HDAC1, Sigma) antibodies. Before use, protein G (Pierce) was blocked with 1 µg/µL sheared herring sperm DNA and 1 µg/µL BSA for 3 h at 4°C and then incubated with chromatin and antibodies for 2 h at 4°C. PCR was performed with HOT-MASTER Taq (Eppendorf) using 2 µL of immuniprecipitated DNA and promoter-specific primers for human *HIF-1α*, *Puma*, *DR5*, and *GAPDH* promoters. Immunoprecipitation with non-specific immunoglobulins (IgG; Santa Cruz Biotechnology) was performed as negative controls. The amount of precipitated chromatin measured in each PCR was normalized with the amount of chromatin present in the input of each immunoprecipitation. PCR products were run on a 2% agarose gel and visualized by ethidium bromide staining using UV light. Primer sequences are available upon request.

### Transfection, plasmids and transactivation assay

Luciferase activity was measured in RKO cells stably transfected with AIP1-luc reporter (RKO-AIP1-luc) or in 293 and RKO cells transiently transfected using respectively the N,N-bis-(2- hydroxyethyl)-2-amino-ethanesulphonic acid-buffered saline (BBS) version of the calcium phosphate procedure [49] and the cationic polymer LipofectaminePlus method (Invitrogen) according to manufacturers' instructions, with the luciferase reporter gene driven by the p53-dependent promoters Noxa-luc (kindly provided by T. Taniguchi, University of Tokyo, Japan) p53AIP1-luc (kindly provided by H. Arakawa, National Cancer Center, Tokyo, Japan), and p21-luc plasmids. Expression vectors used in this study were: HIPK2-Flag [7] and HIPK2-K1182R-Flag unable to be degraded by MDM2 [23]. The amount of plasmid DNA in each sample was equalized by supplementing with empty vector. Transfection efficiency was normalized with the use of a co-transfected β-galactosidase (β-gal) plasmid. Luciferase activity was assayed on whole cell extract and the luciferase values were normalized to β-galactosidase activity and protein content. At least three independent experiments were performed in duplicate.

### Kinase assay

Kinase assay was performed essentially as described [7]. Briefly, equal amount of total cell extracts of 293 transfected with Flag empty vector or HIPK2-Flag expression vector were immunoprecipitated with monoclonal anti-Flag antibody. Immunocomplexes were incubated in kinase buffer in the presence of 5 µCi [γ-^32^P] ATP and 2 µg myelin basic protein (MBP) as substrate, for 30 min at 30°C. Reaction products were resolved by SDS-PAGE and [γ-^32^P]-labeled proteins were detected by autoradiography. Gels were then hydrated and stained with Comassie for protein detection.

### siRNA interference

Cells were plated at semiconfluence in 35 mm dishes the day before transfection. Control-siRNA and siMDM2 (Dharmacon) were transfected overnight using LipofectaminePlus reagent (Invitrogen) and 24 h later cells were treated with CoCl_2_ and Adriamycin for the indicated period before harvesting for Western immunoblotting and luciferase activity.

### *In vivo* tumorigenicity assay

Six-week-old CD-1 nude (nu/nu) mice (Charles River Laboratories) were used for *in vivo* studies. They were housed in specific pathogen-free conditions and fed standard cow pellets and water ad libitum. Studies were performed in accordance with institutional standard guidelines for animal experiments. Solid tumors were obtained by injecting i.m. on the flank of each mouse 7x10^6^ viable RKO cells suspended in 0.1 mL PBS. The mice were examined every day after injection until tumors reached approximately 300 mm^3^ weight (7 days from injection). Mice were then randomized in four groups (6–8 mice/group) as follow: 1) ADR (10 mg/kg body weight), 2) ZnCl_2_ (10 mg zinc/kg body weight), 3) combination of ADR plus ZnCl_2_, and 4) PBS. At day 7, mice were pre-treated with ZnCl_2_ for 8 h and then ADR was injected once i.p.; subsequently, ZnCl_2_ was administrated once daily by oral administration, over the course of two weeks. Tumor dimensions were measured every other day and their volumes were calculated from caliper measurements of two orthogonal diameters (*x* and *y*, larger and smaller diameters, respectively) by using the formula volume = xy^2^/2. The antitumor effect of the combination treatment, zinc+ADR, was evaluated by comparing the relative tumor size with tumors treated with ADR only or zinc only. The experiment was repeated twice with similar results.

### Ethics Statement

All animals were handled in strict accordance with good animal practice as defined by the relevant national and/or local animal welfare bodies, and all work was performed in accordance with the guidelines of the National Cancer Institute Regina Elena, and in accordance with the Italian legislation.

### Statistics

All experiment unless indicated were performed at least three times. All experimental results were expressed as the arithmetic mean and standard deviation (s.d.) of measurements was shown. Student's *t*-test was used for statistical significance of the differences between treatment groups. Statistical analysis was performed using analysis of variance at 5% (p<0.05) or 1% (p<0.01).
